# An early HMGB1 rise 12 hours before creatinine predicts acute kidney injury and multiple organ failure in a smoke inhalation and burn swine model

**DOI:** 10.3389/fimmu.2024.1447597

**Published:** 2024-10-29

**Authors:** Zhangsheng Yang, Tomas S. Cancio, Robert P. Willis, Matthew D. Young, Dustin M. Kneifel, Jose Salinas, Andrew D. Meyer

**Affiliations:** ^1^ Organ Support and Automation Technologies, United States Army Institute of Surgical Research, Fort Sam Houston, TX, United States; ^2^ Long School of Medicine, University of Texas Health Science Center, San Antonio, TX, United States

**Keywords:** high-mobility group box 1, acute kidney injury, multiple organ failure, swine, polytrauma, inflammation

## Abstract

**Background:**

Acute kidney injury (AKI) and multiple organ failure (MOF) are leading causes of mortality in trauma injuries. Early diagnosis of AKI and MOF is vital to improve outcomes, but current diagnostic criteria rely on laboratory markers that are delayed or unreliable. In this study, we investigated whether damage associated molecular patterns such as high-mobility group box 1 (HMGB1), syndecan-1 (SDC-1) and C3a correlate with the development of trauma-induced AKI and MOF.

**Methods:**

Thirty-nine swine underwent smoke inhalation and severe burns, then received critical care for 72 hours or until death. AKI was defined by the KDIGO (Kidney Disease: Improving Global Outcomes) criteria, which labels AKI when a 1.5-fold increase in blood creatinine levels from baseline or a urine output < 0.5 mL/kg/h for 6 hours or more occurs. MOF was defined by the presence of both AKI and acute respiratory distress syndrome (PaO_2_/FiO_2_<300 for 4 hours).

**Results:**

Eight of 39 pigs developed AKI and seven of those developed MOF. Pathological analysis revealed that polytrauma induces significantly higher kidney injury scores compared to sham controls. The average time from injury to KDIGO AKI was 24 hours (interquartile range: 22.50-32.25). Twelve hours after injury, HMGB1 levels were significantly increased in animals that went on to develop AKI compared to those that did not (73.07 ± 18.66 ng/mL vs. 31.64 ± 4.15 ng/mL, *p*<0.01), as well as in animals that developed MOF compared to those that did not (81.52±19.68 ng/mL vs. 31.19 ± 3.972 ng/mL, *p*<0.05). SDC-1 and C3a levels were not significantly different at any time point between groups. ROC analysis revealed that HMGB1 levels at 12 hours post-injury were predictive of both AKI and MOF development (AKI: AUROC=0.81, cut-off value=36.41 ng/mL; MOF: AUROC=0.89, cut-off value=36.41 ng/mL). Spearman’s correlation revealed that HMGB1 levels at 12 hours correlated with multiple parameters of AKI, including blood urea nitrogen, blood creatinine, and blood myoglobin.

**Conclusion:**

Twelve-hour post-injury HMGB1 levels predict AKI and MOF in a smoke inhalation and burn swine model. Further research is needed to validate this result in other polytrauma models and in critical combat causalities.

## 1 Introduction

Severe smoke inhalation and burn injury is known to cause acute kidney injury (AKI), multiple organ failure (MOF), and death (1, 2). Trauma patients that develop AKI are known to have three times higher mortality rate then trauma patients that do not have AKI (3). Trauma-induced MOF can have up to a 50% mortality rate in polytrauma patients (4). In battlefield environments with critically ill combat causalities, over 34% develop AKI (5), and 20% develop MOF (6). Several risk factors contribute to the development of trauma-induced AKI including hypotension, hemolysis, smoke inhalation, and burn-induced systemic inflammation and apoptosis (7). Earlier studies have shown that trauma injuries can activate the innate immune system to release of damage-associated molecular patterns (DAMPs) and/or pathogen associated molecular patterns (PAMPs) that exacerbate inflammatory kidney damage (8).

Clinically, AKI is defined by either a prolonged increase in serum creatinine levels (1.5 or more times baseline) or development of acute oliguria (decreased in urinary output of less than 0.5 mL/kg/h for 6-12 h) (9). MOF is defined by failure of more than one organ system. Several scoring systems have been developed for the diagnosis of MOF. The most widely used being the Denver MOF score, the Sequential Organ Failure Assessment (SOFA), and the Multiple Organ Dysfunction Score (MODS) (10). MOF criteria are met when either 1) two of six vital organ systems (the respiratory, renal, hepatic, cardiovascular, hematologic, and neurologic) fail and require medical intervention or 2) when a certain level of dysfunction is reached across all six of these systems according to a scored numeric scale (11). Importantly, the number of failing organ systems directly correlates with increased mortality rates. Failure of two vital organ systems results in a 32% increase in mortality which further increases significantly with the failure of each additional organ system (12). Studies have highlighted the importance of systemic inflammation cannot be understated in contributing to MOF, as it has been documented to release PAMPs and DAMPs that cause inflammation and organ damage throughout the body.

Lack of early diagnostic tools in current clinical practice often results in a late diagnosis of AKI and MOF causing delayed treatment, suboptimal care, and poor patient outcomes. Therefore, there is a critical need for diagnostic tools that provide an earlier indication of AKI and MOF. Earlier research showed that neutrophil gelatinase-associated lipocalin (NGAL) to be most associated with early diagnosis of AKI (13, 14). Clinical applicability of other biomarkers such as N-acetyl-β-glucosaminidase (NAG) (15), interleukin 18 (IL-18) (16), and kidney injury molecule-1 (KIM-1) (17) have remained theoretical. As of 2020, the Acute Disease Quality Initiative Consensus Conference argued that there is an urgent need for the exploration of novel biomarkers for early diagnosis of AKI, as the current clinical guidelines rely on delayed methods (18). The use of biomarkers to diagnose MOF is also vital to prioritize resources and interventions. Recent advancement in the use of specific biomarkers, such as procalcitonin for sepsis, has led to significant personalization of care reducing waste and side effects for unneeded treatments (19).

Circulating HMGB1 levels are known to increase significantly within hours post-injury (20). Extracellular HMGB1 binds to toll-like receptors (TLRs) or the Receptor for Advanced Glycation End-products (RAGE), exacerbating the inflammatory process by encouraging cytokine release leading to organ failure (21). Our group has previously shown that early increases of HMGB1 after polytrauma can predict organ failure such as acute respiratory distress syndrome (ARDS) (21). Additionally, endothelial dysfunction has been shown to worsen the severity of AKI in the clinical setting (22) and has been associated with early organ impairment (23). SDC-1 is a specific biomarker that releasing from damaged endothelial cells and can therefore be an important alarmin for vascular damage (24, 25). Moreover, a proinflammatory complement component (C3a) has been shown to rise significantly after injury and is associated with mortality (26, 27). Our hypothesis for this study was to determine if increases in DAMPs that include high-mobility group box 1 (HMGB1), syndecan-1 (SDC-1), and C3a correlate with an early diagnosis of AKI or MOF in smoke-inhalation and burn injury model.

## 2 Materials and methods

**Figure 1:**
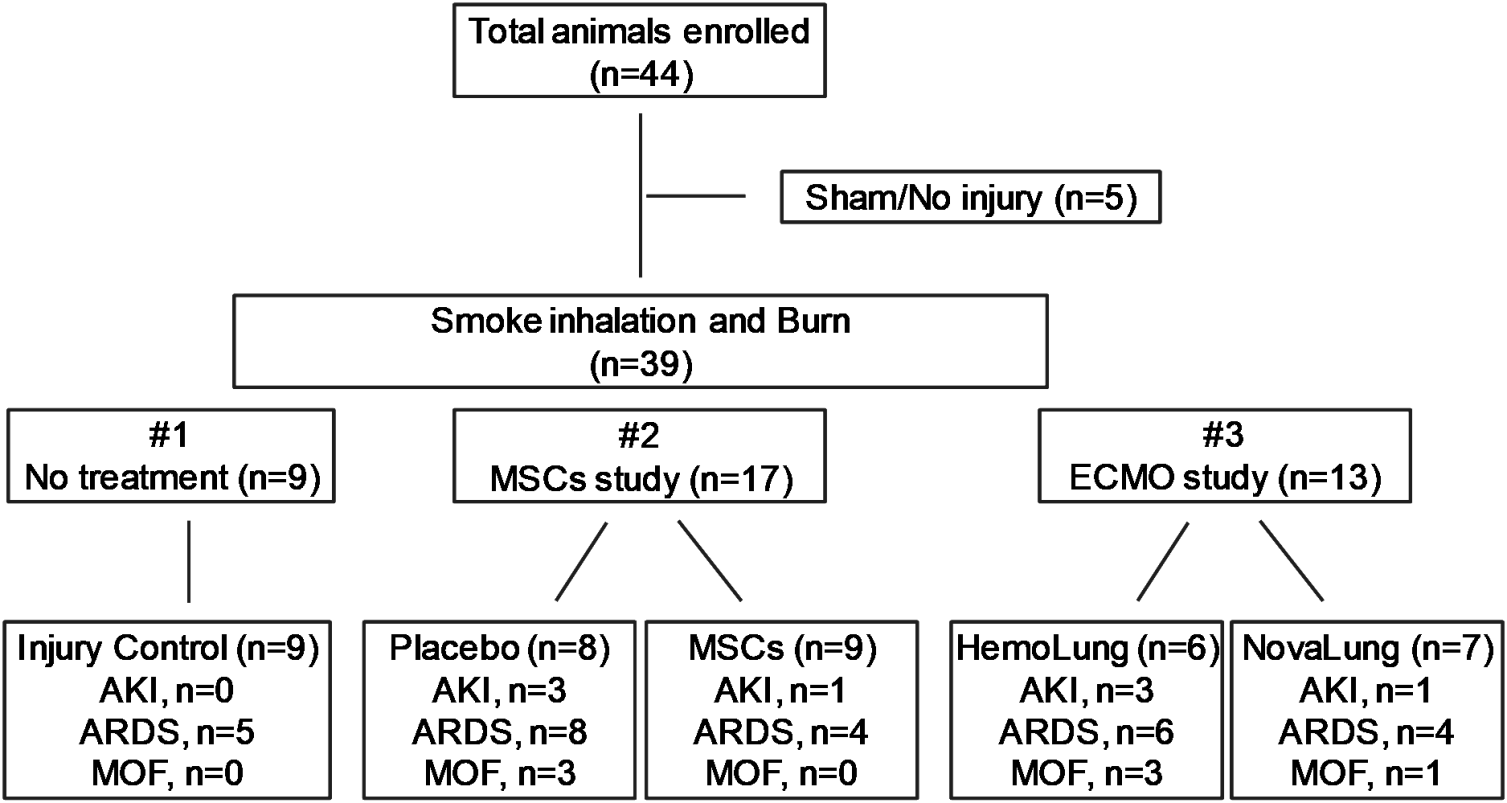
The flow chart of experimental design and animal groups. Three individual studies including injury control (n=9), MSCs study (n=17) and ECMO study (n=13) were enrolled, and the distributions of AKI, ARDS and MOF in each experiment were shown.

Data and samples for this study was obtained from three independent experiments (**Figure 1**). All studies were approved by the Institutional Animal Care and Use Committee (IACUC) of the U. S. Army Institute of Surgical Research (USAISR). Research was conducted in compliance with the Animal Welfare Act, Animal Welfare regulations, and the principles of the Guide for the Care and Use of Laboratory Animals. IACUC reviewed and approved all research conducted in this study. The facility where this research was conducted is fully accredited by AAALAC International.

### 2.1 Animal preparation

All studies followed an established model at USAISR for smoke inhalation and burn injury as previously described (28, 29). Non-pregnant female Yorkshire swine weighing 35-50 kg received standard surgical preparation, were premedicated with Telazol (tiletamine/zolazepam 6 mg/kg), glycopyrrolate (0.01 mg/kg), and anesthetized with isoflurane. Next, the animals were cleaned, shaved and intubated with an 8-10 French (Fr.) endotracheal tube as well as receiving a Foley catheter. Under sterile conditions, central lines (8–8.5-Fr catheters, Arrow Int’l, Reading, PA) were placed in the bilateral jugular and femoral veins and bilateral femoral arteries using a modified Seldinger technique (30). Further, an open tracheostomy via direct laryngoscopy was performed and, following successful placement of a central venous line, total intravenous anesthesia (TIVA) was initiated using midazolam (5-20 mcg/kg/min), fentanyl (0.01-0.1 mcg/kg/min), ketamine (20-30 mg/kg/hr), and propofol (50-100 µg/kg/min). The animal was then slowly weaned off isoflurane. A femoral line was used for arterial blood pressure monitoring. Once the animal had stabilized, a baseline (BL) sample was taken and the animal underwent smoke inhalation and a 40% TBSA full-thickness burn injury: wood smoke was delivered to induce injury and a full-thickness burn was achieved using an open flame (Bunsen burner) (28). TIVA was titrated as needed and continued throughout the remainder of the study. Proper analgesic control was assured by toe pinching across the coronary band with a large hemostat as well as regular checking of jaw tone to ensure a surgical plane of anesthesia.

### 2.2 Study groups and interventions

In total, 44 animals were analyzed for this study. 39 swine underwent polytrauma with smoke inhalation and burn injury, while five swine were cannulated only without further injury, serving as sham control animals. Data and samples for the 39 animals were obtained from three individual experiments (**Figure 1**). The first study consisted only of injury control animals with no treatment (n=9); the second study included animals with placebo (saline n=8) and autologous mesenchymal stromal cells (MSCs) treatment (MSCs, n=9); and the third study included animals with extra-corporeal life support (ECLS) devices, either the Hemolung (ALung Technologies, Pittsburgh, PA) (n=6) or the NovaLung (Xenios, Heilbronn, Germany) (n=7). In each original experiments, the animals were randomly assigned to different treatment arms. All the treatments to the injured animals were performed right after smoke inhalation and burn injury. Once interventions were completed, the animals were moved to the Intensive Care Unit (ICU) and were held under close observation for up to 72 hours or until death. All animals received mechanical ventilation (MV) with a bilevel (BL) mechanical ventilation of 10 mL/kg tidal volume (TV) in all groups. In accordance with standard of care practice, TV was titrated as ARDS developed using the ARDSnet protocol to maintain normocarbia (31, 32). MSCs were harvested from the iliac crest of and femur of the swine. Collected bone marrow was concentrated using a bedside cell concentrator (Magellan, Arteriocyte, Hopkinton, MA) as previously described (31). Next, the MSC’s were suspended with Plasma-Lyte to a working volume of 60 mL and administered over a 30-minute time period for three doses, taking place at 6-, 24-, and 48-hours post-injury. Animals received a dose of MSCs sufficient to contain approximately 5.66 × 10^7^ platelets/kg (33).

For the extracorporeal life-support (ECLS) treatment animals, either the Hemolung or the Novalung ECLS device was implemented based on their study group. Both systems were primed with 0.9% normal saline provided with the respective ECLS devices and received an initial bolus of 5,000 units of unfractionated heparin plus a continuous infusion of heparin to maintain an activated clotting time (ACT) above 50 s. Ventilator settings were reduced as much as possible to reduce peak inspiratory pressure (PIP) while maintaining normocarbia.

### 2.3 Vital sign, blood gas, biochemistry and blood sample collection

Using a high-pressure monitoring line (Smith Medical ASD Inc., Dublin, OH) connected to the femoral artery, vital signs, including heart rate and arterial blood pressure, were tracked, recorded, and stored using proprietary data acquisition software (Integrated Data Exchange and Archival [IDEA] system) as previously described (34, 35). The urine output data was collected and recorded on an hourly basis. A pulmonary artery catheter was placed via a jugular vein to permit measurement of cardiac output by thermodilution and pulmonary artery pressures. The blood gases were analyzed using a bedside iSTAT 300-G blood analyzer (Abbott Point of Care Inc., Princeton, NJ; VetScan CG4+ and CG8+ cartridges, Abaxis Inc., Union City, CA). Blood chemistry of myoglobin was analyzed using Dimension Xpand Plus Integrated Chemistry System (Siemens, Holliston, MA) by a chemistry laboratory in the USAISR (34). To analyze the inflammatory mediators in the circulation blood, serum was drawn at BL, post-injury, and 1-, 3-, 6-, 12-, 24-, 48-, and 72-hours post-injury, or at death in the case of early death. After drawing, the blood samples were processed, aliquoted, and stored at −80°C for later analysis. Lastly, tissue samples were collected during necropsy and were processed for histological use and immunohistochemistry (IHC) assays.

### 2.4 Measurement of inflammatory mediators by ELISA

Quantitative analysis of inflammatory mediators HMGB1, SDC-1, and C3a in serum samples was performed as previously described (21). Enzyme-linked immunosorbent assay (ELISA) kits for analyzing HMGB1 (IBL International, Cat# 30164033), SDC-1 (Cloud-Clone Corporation, Cat# SEB966Po), and C3a (MyBioSource, Cat# MBS2509360) were purchased from commercial companies. The quantitative measurements were performed according to the manufacturer’s recommendations.

### 2.5 Histology evaluation and immunohistochemistry assay

The kidney tissue was collected and processed for histology and IHC staining as described previously (36). The tissues were fixed in 10% formalin and were embedded in paraffin. Coronal sections were then cut and stained with hematoxylin-eosin (H&E). Thirty random histologic images were recorded with a 10× objective under a slide scanner (Axio Scan. Z1 v1.0, Zeiss, Germany), and scored by the pathologist blind to the ID of animal under magnification=400×. The changes were semi-quantitatively scored according to the extent of injury (score 0, 1, 2, 3, and 4 for an extent of 0%, < 25%, 25–50%, 50–75%, and 75–100%, respectively) and the severity of injury: score 0 = normal histology; score 1 = slight alteration (loss of brush border, mild hydropic degeneration, mild congestion); 2 = mild (intensive hydropic degeneration, mild vacuolization, and interstitial edema); 3 = moderate (nuclear condensation, intensive vacuolization, modulate interstitial edema); and 4 = severe (necrotic/apoptotic cells, denudation/rupture of basement membrane). The injury score represents the sum of the scores for the extent and severity of the injury (34).

The cleaved caspase-3 in the tissue was analyzed for IHC evaluation of cellular apoptosis and organ damage. The samples were collected and fixed in 10% neutral buffered formalin and processed for paraffin embedding. Tissue was sectioned (4 µm) then cleared in xylene and rehydrated using 100% ethanol, 95% ethanol 70% ethanol and DI water. Heat-induced antigen retrieval was performed in citrate buffer (pH 6.0) for 20 minutes at 95˚C. Staining was performed by blocking with horse serum with 0.1% Triton X-100, followed by application of primary antibody of rabbit anti-cleaved caspase-3 (1:500 dilution, Cat# 9661, Cell signaling) for 30 minutes. After incubation, slides were rinsed and biotinylated anti Rabbit secondary antibody was applied (1:250 dilution, Cat# BA-2000, Vector Labs) for 30 minutes. DAB horseradish peroxidase chromogen (Cat# SK-4105, Vector labs) was used to develop the antibody. All slides were counterstained with hematoxylin and cleared for cover slipping.

### 2.6 Statistical analysis

**Figure 2:**
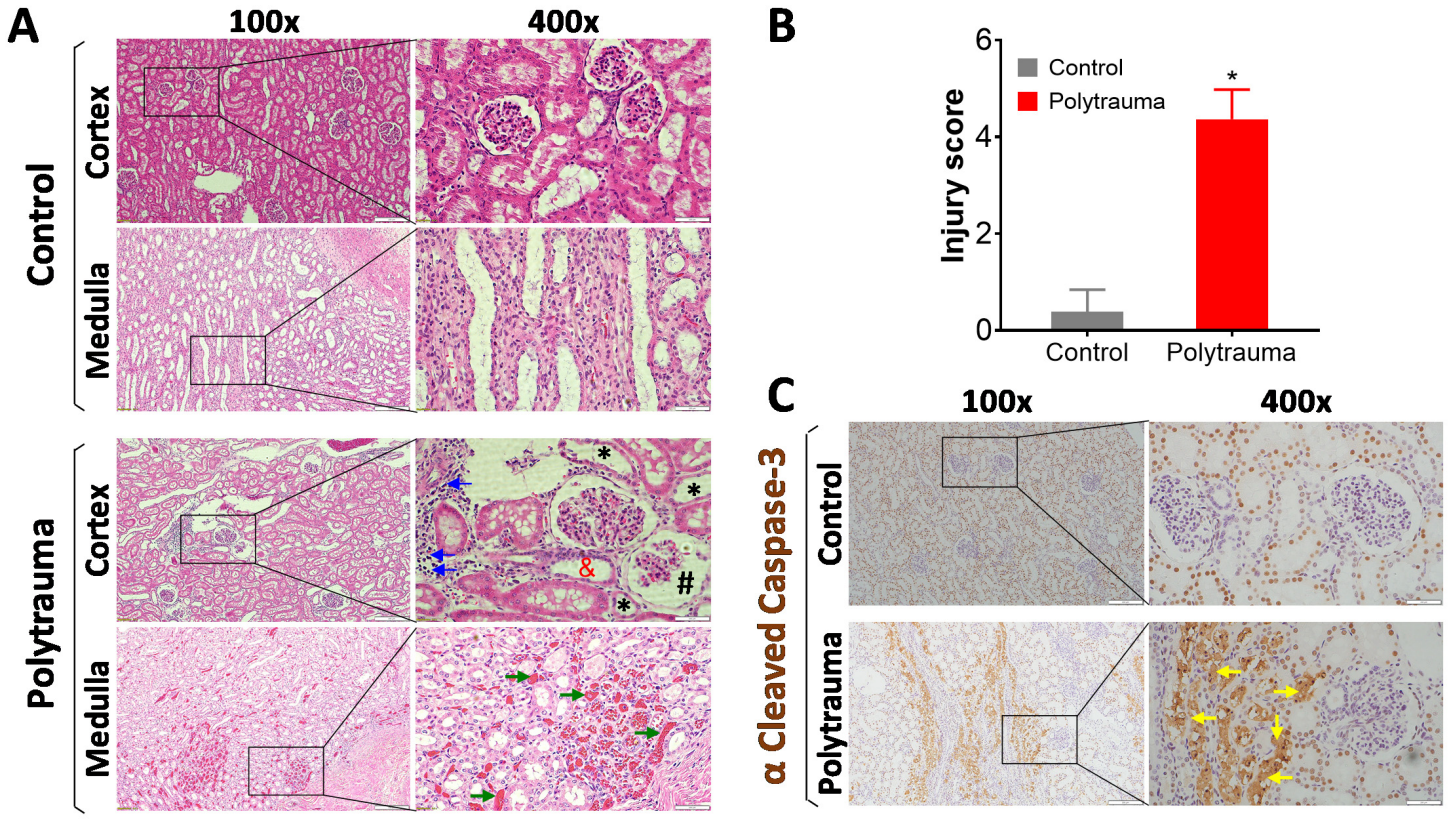
Histological and IHC detection of inflammation, cell death and tissue damage in control and injured swine. The swine underwent smoke inhalation and 40% total body surface area (TBSA) burn injury, or cannulation only as the sham control, followed up observation in the ICU care for up to 72 hours post-injury or unless early death. **(A)** Representative images of histological evaluation of kidney tissue in the cortex and medulla area in the control swine and polytrauma injured swine. In the injured animals, lots of inflammatory infiltration (blue arrows), epithelial thinning (black star), proximal tubular dilation (red and), glomerulus Bowman’s spacing (black pound) were detected in the cortex of kidney, and severe hemorrhage/thrombosis (green arrow) was detected at medulla area. **(B)** semi-quantitative scoring of kidney injury in the control and injured animals. The data are presented as mean ± SD; *, *p*<0.05, performed by Mann-Whitney U test. n=4 for control, and n=5 for injured animals. **(C)** Representative images of IHC staining of cleaved caspase-3 in the control and injured swine kidney in the cortex area. The depositions of cleaved caspase-3 were indicated by yellow arrows. For all the images, the magnification is 100 × for the left panels, and 400 × for the right panels. Scale bar is 200 µm in each individual image.

Chemistry, vital signs and blood gas data in **Table 1** and organ injury score in **Figure 2** were presented as mean and standard deviation (SD), and a Mann–Whitney U test followed by a Dunn’s *post-hoc* test was applied for the statistical analyses. Longitudinal data were presented as mean and standard error of the mean (SEM). Some of the data points were absent in the longitudinal data thus a mixed-effect model for repeated measures with random intercept and random slope or a Friedman test followed by a Dunnett’s or Dunn’s *post-hoc* test (where appropriate) was used to examine within-group-specific differences in defined HMGB1, SDC-1, and C3a biomarkers throughout the observation period after injury, and each time point by the groups of AKI and non-AKI (or MOF vs non-MOF) as fixed effects. Receiver operating characteristic (ROC) curves were plotted for HMGB1, SDC-1, C3a, mean arterial pressure (MAP), PCO2, and oxygen saturation (SPO2) for predicting likelihood of AKI or MOF for all conditions at 12-hours post-injury. The optimal cutoff values with the Youden index and the areas under the ROC curves (AUROC) were calculated. Sensitivity and specificity using the optimal cutoff values for predicting outcomes were also performed. Logistic regression analysis was used for calculating odds ratios (ORs) with 95% confidence intervals (95% CI) for clinical outcomes of AKI and MOF. Statistical significance was determined with a two-sided p < 0.05. Statistical analyses were performed using GraphPad Prism 9.3.1 (GraphPad Software, San Diego, CA).

**Table 1:** The chemistry, blood gas, and vital sign data of animals underwent smoke inhalation and burn injury.

Parameters	Groups	BL	PI	1 hr	3 hr	6 hr	12 hr	24 hr	48 hr	72 hr PI	Reference ranges*
Myoglobin (mcg/L)	Non-AKI (n=31)	59.53 ± 30.62	n/a	n/a	n/a	n/a	n/a	393.53 ± 301.34	519.73 ± 377.18	408.69 ± 389.14	5-70 mcg/L (37)
	AKI (n=8)	61.28 ± 30.51	n/a	n/a	n/a	n/a	n/a	1405.57 ± 1077.48**	n/a	397.00 ± 0	
BE/BD (mmol/L)	Non-AKI (n=31)	7.54 ± 3.91	6.86 ± 4.87	5.48 ± 4.46	5.05 ± 3.48	4.24 ± 2.82	2.72 ± 2.16	3.82 ± 3.85	6.89 ± 3.68	11.06 ± 15.70	-4 - +2 (37)
	AKI (n=8)	4.63 ± 2.77	8.13 ± 4.12	3.25 ± 2.50	3.86 ± 2.34	3.25 ± 3.66	0.13 ± 4.91	1.00 ± 5.93	3.00 ± 0	2.00 ± 0	
Lactate (mmol/L)	Non-AKI (n=31)	5.56 ± 16.35	3.48 ± 2.86	3.02 ± 3.09	2.05 ± 2.01	1.63 ± 1.97	0.80 ± 0.26	1.10 ± 1.42	0.87 ± 0.70	1.29 ± 2.16	0.5-5.5 (38)
	AKI (n=8)	2.01 ± 0.87	2.63 ± 0.92	2.07 ± 0.66	1.78 ± 0.64	1.40 ± 0.77	1.91 ± 2.46	2.62 ± 2.20	0.40 ± 0	0.31 ± 0	
Glucose (mg/dL)	Non-AKI (n=31)	130.07 ± 37.05	201.40 ± 59.23	n/a	n/a	94.54 ± 23.42	91.79 ± 17.98	91.20 ± 35.47	82.56 ± 33.07	66.96 ± 24.81	52-153.88 (39)
	AKI (n=8)	135.13 ± 19.25	235.88 ± 48.77	n/a	n/a	96.43 ± 24.10	84.00 ± 13.08	116.50 ± 44.86	116.00 ± 0	56.00 ± 0	
K^+^(mEq/L)	Non-AKI (n=31)	3.47 ± 0.34	4.79 ± 0.81	n/a	n/a	4.43 ± 0.45	4.18 ± 0.44	3.81 ± 0.32	3.79 ± 0.46	4.04 ± 0.47	3.5-4.7 (39)
	AKI (n=8)	3.51 ± 0.24	5.0 ± 1.19	n/a	n/a	4.61 ± 1.01	4.25 ± 0.53	4.66 ± 1.78*	3.50 ± 0	4.10 ± 0	
Na^+^ (mEq/L)	Non-AKI (n=31)	138.21 ± 1.83	132.57 ± 2.77	n/a	n/a	136.14 ± 3.12	138.00 ± 3.81	139.14 ± 4.32	142.73 ± 5.37	145.52 ± 7.12	129-142.75 (39)
	AKI (n=8)	138.63 ± 1.92	131.50 ± 2.93	n/a	n/a	135.57 ± 1.81	139.00 ± 3.59	139.71 ± 4.39	146.00 ± 0	150.00 ± 0	
Cardiac output (Cont.)	Non-AKI (n=31)	5.59 ± 1.29	3.54 ± 0.98	3.28 ± 0.48	3.33 ± 0.36	3.05 ± 0.75	3.31 ± 0.88	4.17 ± 0.86	5.45 ± 0.73	6.33 ± 0.95	3.4-7.4 (40)
	AKI (n=8)	6.13 ± 1.47	3.07 ± 0.48	2.98 ± 0.59	3.08 ± 1.01	2.79 ± 0.78	3.23 ± 1.22	3.65 ± 1.34	n/a	5.90 ± 0	
Shock Index	Non-AKI (n=31)	0.80 ± 0.19	0.81 ± 0.27	0.73 ± 0.22	0.71 ± 0.27	0.69 ± 0.21	0.80 ± 0.22	0.94 ± 0.19	1.00 ± 0.22	1.03 ± 0.25	0.83 ± 0.10 (41)^&^
	AKI (n=8)	0.88 ± 0.24	0.94 ± 0.41	0.97 ± 0.61	0.98 ± 0.49	0.96 ± 0.33	0.96 ± 0.37	1.14 ± 0.36	0.94 ± 0	0.88 ± 0	
PFR	Non-AKI (n=31)	447.95 ± 38.54	449.60 ± 51.51	443.48 ± 133.60	396.76 ± 90.95	399.73 ± 81.90	393.37 ± 83.97	325.50 ± 131.03	294.18 ± 123.11	238.89 ± 124.95	>300 (42)^$^
	AKI (n=8)	439.88 ± 30.34	417.88 ± 72.86	331.67 ± 27.06	385.43 ± 63.56	346.79 ± 72.72	327.21 ± 141.58	248.67 ± 118.51	316.00 ± 0	324.00 ± 0	

BL, baseline; PI, post-injury; BE/BD, base excess/base deficit; PFR, PaO2/FiO2 Ratio. Data were presented as mean ± SD, and statistical analyses were performed by Mann-Whitney U test. *, p<0.05, **, p<0.01, Non-AKI vs. AKI. Significant differences are indicated by boldface type. ^&^Shock index was calculated from normal physiological parameters in swine used for biomedical research as established by Hannon JP, et al. (41). ^$^PFR reference range was based off clinical criteria established in human populations.

## 3 Results

### 3.1 Polytrauma caused AKI and MOF in injured animals

Among the 39 injured animals, eight (20.5%) developed AKI (**Figure 1**), with a median time of 24 hours (interquartile ranges, 22.50-32.25) post-injury. Four were detected in MSCs study with three in the saline arm (n=3/8) and one in treatment arm (n=1/9). The other four come from ECMO support study with the Hemolung arm (n=3/6) and Novalung arm (n=1/7). AKI did not develop in the other 31 (79.5%) animals over the 72-hour period of observation in the ICU. Seven (17.9%) swine with AKI also developed ARDS and were classified as MOF (development of both AKI and ARDS). Three and four of the MOF swine were from the MSC and ECMO studies, respectively. The other 32 (82.1%) pigs developed AKI only, ARDS only, or neither AKI nor ARDS (**Figure 1**).

Histological evaluation showed that severe tissue damage presented in the cortex and medulla area of the injured animals (**Figure 2A**). To compare the overall histological differences between the injured animals and sham controls, a semi-quantitatively calculation was performed. Data showed, significantly higher injury scores were observed in polytrauma swine compared to control animals (**Figure 2B**). Furthermore, IHC staining detected high levels of cleaved caspase-3 in the injured animals (**Figure 2C**).

### 3.2 AKI and blood gas parameters showed significant differences late or not at all

**Figure 3:**
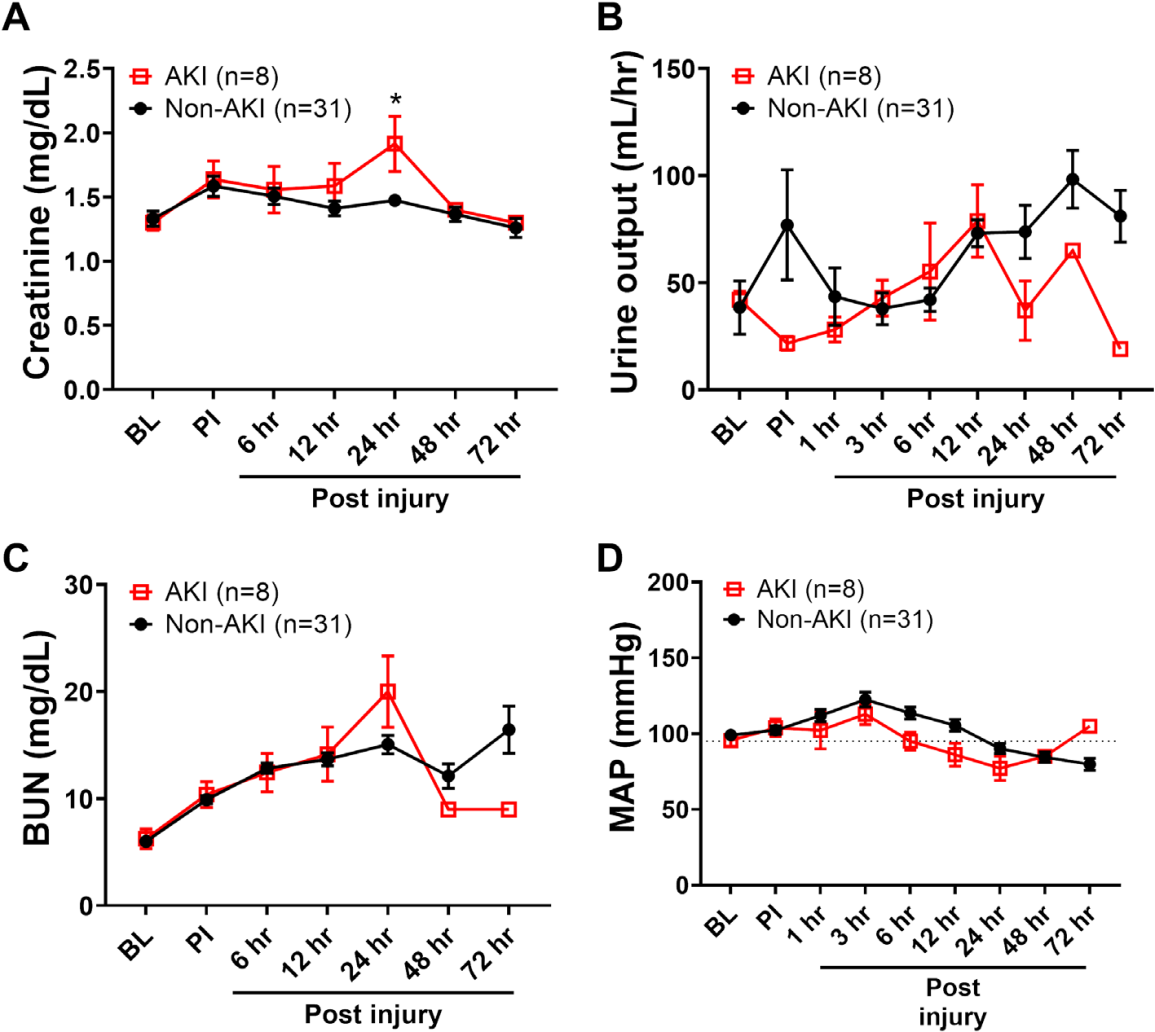
The clinical variables of animals underwent smoke inhalation and burn injury. The dynamic changes of blood creatinine **(A)**, urine output **(B)**, blood urine nitrogen (BUN, **C**) and MAP **(D)** in the swine with AKI vs swine without AKI (Non-AKI) were recorded for up to 72 hours post-injury and presented. Data are presented as mean ± SEM and were statistically analyzed using mixed-effects model for repeated measures. *, p<0.05. The normal physiological ranges for MAP is indicated by dotted lines.

Comparing the standard AKI parameters between the swine with AKI and those that did not develop AKI (non-AKI) reveal that the differences are relatively minor and late in relation to disease progression. The blood creatinine level was only statistically significant higher at 24 hours post-injury in AKI swine compared to non-AKI swine (**Figure 3A**). The urine output and blood urine nitrogen (BUN) were not significantly different among the time points observed (**Figures 3B, C**). The MAP was not significantly different comparing these two groups, although it is notably lower in AKI animals (**Figure 3D**). The myoglobin and potassium levels are significantly higher at 24 hours in AKI swine compared to non-AKI swine (**Table 1**). No statistical difference was observed for base excess/base deficit (BE/BD), lactate, glucose, sodium, cardiac output, shock index, and PaO2/FiO2 Ratio (PFR) at any given time point from baseline to 72 hours post-injury (**Table 1**).

### 3.3 HMGB1 levels were significantly increased in the AKI animals compared to non-AKI injured animals

**Figure 4:**
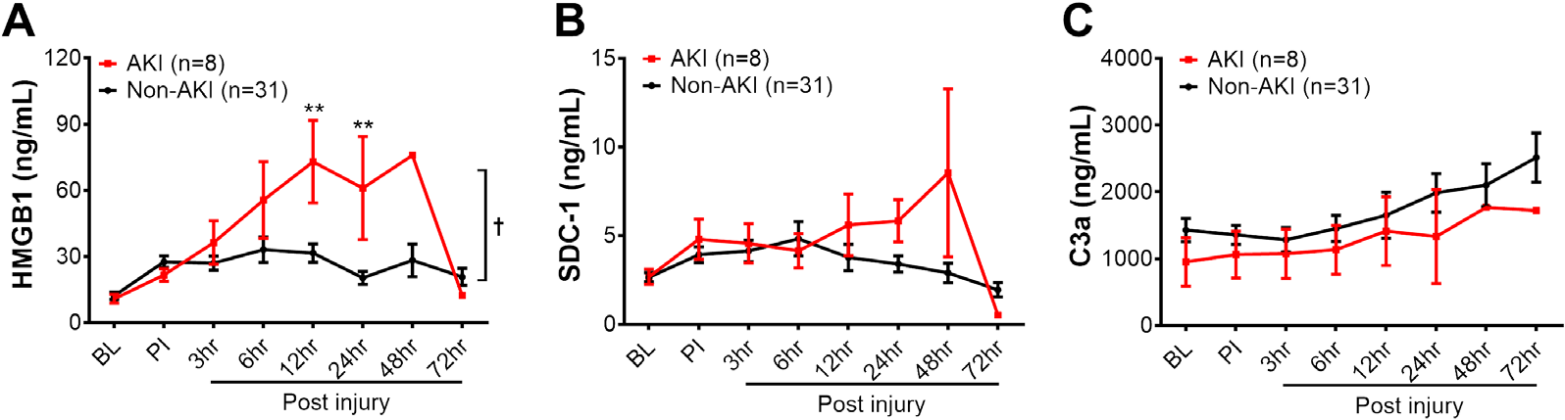
The dynamic changes of biomarkers in the blood circulation of animals in AKI and non-AKI groups. The levels of HMGB1 **(A)**, SDC-1 **(B)**, and C3a **(C)** in the swine of AKI (n=8) vs. non-AKI (n=31) in the serum were measured by ELISA, including baseline (BL), post-injury (PI), and 3, 6, 12, 24, 48, and 72 hours post-injury. The data were presented as mean ± SEM. Statistical analyses were performed by linear mixed-effect model for repeated measures, and for the least square means of individual group comparisons. **, p<0.01, the value of individual time point of swine with AKI vs. non-AKI; and †, p<0.05, for the least square means between the groups.

Next, we evaluated three inflammatory mediators in the blood, including HMGB1, a typical DAMP involved in cell damage/injury and stress; SDC-1, a biomarker indicating endothelial cell damage; and complement factor C3a, an anaphylatoxin that can attract inflammatory cells, mediating profound proinflammatory signal activation. The HMGB1 levels were significantly increased in the AKI group at individual 12-, 24-hours post-injury, and overall when compared to non-AKI animals (**Figure 4A**). The SDC-1 and C3a levels were not significantly different between pigs with AKI and without AKI (**Figures 4B, C**). Subsequently, the association of SDC-1 and C3a at 12 hours PI to AKI outcome were not significant (**Table 2**).

**Table 2:** ROC analysis of risk factors associated with AKI and MOF at 12 hours post-injury.

ROC	AUROC	*p-*value	Cut-off	Sensitivity	Specificity
SDC-1 with AKI	0.68	0.17	n/a	n/a	n/a
C3a with AKI	0.54	0.77	n/a	n/a	n/a
Creatinine with AKI	0.62	0.35	n/a	n/a	n/a
Urine Output	0.54	0.73	n/a	n/a	n/a
MAP with AKI	0.76	0.02	100 mmgHg	75.00%	66.67%
SDC-1 with MOF	0.74	0.08	n/a	n/a	n/a
C3a with MOF	0.58	0.58	n/a	n/a	n/a
Creatinine with MOF	0.56	0.66	n/a	n/a	n/a
Urine output with MOF	0.63	0.32	n/a	n/a	n/a
MAP with MOF	0.77	0.03	100 mmgHg	71.43%	64.52%

ROC, receiver operating characteristic; AUROC, area under the receiver operating characteristic; SDC-1, syndecan-1; MAP, mean arterial pressure; n/a, not applicable.

The same outcome was observed in pigs with and without MOF development (**Supplementary Figure 1**). At 12 hours post-injury, HMGB1 levels were significantly elevated in pigs with MOF compared to those without MOF (**Supplementary Figure 1A**). No statistical difference was observed for SDC-1 and C3a among MOF and non-MOF swine during the 72-hour follow up period. The association of SDC-1 and C3a to MOF outcomes was not significant, either (**Table 2**).

### 3.4 HMGB1 was correlated with AKI parameters in injured animals

**Figure 5:**
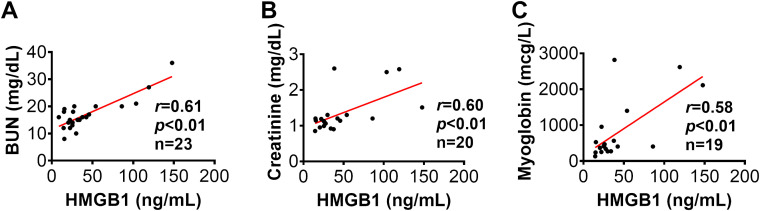
The correlation of HMGB1 to clinical variables in injured swine with smoke inhalation and burn injury. The correlations of HMGB1 at 12 hours post-injury with blood urine nitrogen [BUN, panel **(A)**], blood creatinine **(B)** and myoglobin **(C)** at 24 hours post-injury were performed using Spearman’s rank correlation, and the data are presented with a coefficient (r_s_) and *p*-values. Significant correlations (*p <*0.05) are indicated by boldface type.

Furthermore, Spearman’s correlation analysis showed that the HMGB1 levels at 12 hours post-injury was significantly correlated with several AKI parameters, including BUN (*r*=0.61 *p*<0.01), creatinine (*r*=0.60, *p*<0.01), and myoglobin (*r*=0.58, *p*<0.01), thus indicating HMGB1 elevation correlates to clinical outcome metrics of AKI in animals subjected to smoke inhalation and burn injury (**Figure 5**). In addition, Spearman’s correlation analysis revealed that the level of creatinine is positively correlated with the level of myoglobin in the injured animals with smoke inhalation and burn injury (**Supplementary Figure 2**).

### 3.5 HMGB1 predicted AKI and MOF in injured animals

**Figure 6:**
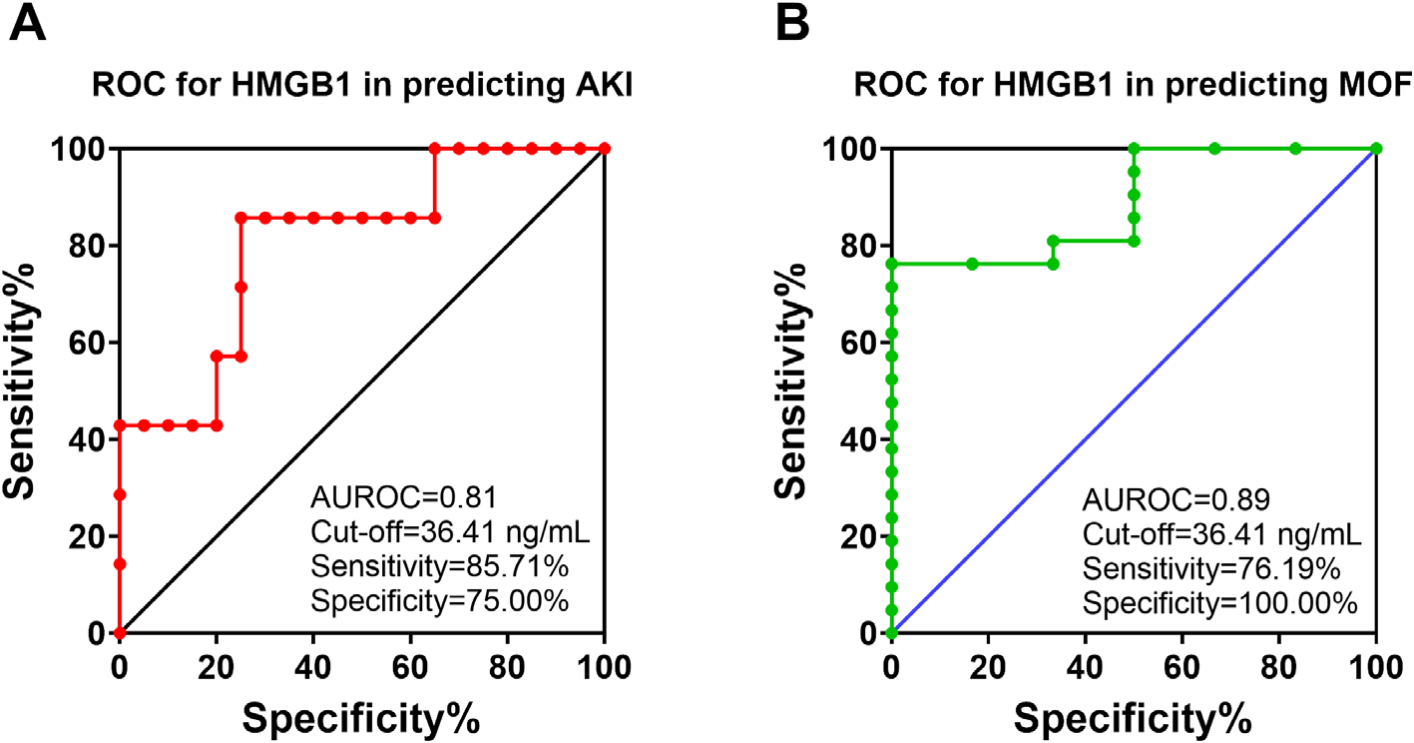
The receiver operating characteristic (ROC) analysis of HMGB1 with AKI and MOF development in animals with polytrauma. The ROC curve for analyzing of HMGB1 in the serum (12 hours post-injury) in predicting of AKI **(A)** and MOF **(B)** in animals underwent polytrauma of smoke inhalation and burn injury. The area under the ROC (AUROC), the optimal cut-off value with Youden index, sensitivity and specificity for each prediction are listed in the right-side panel of each graph.

ROC analysis revealed that HMGB1 levels at 12 hours post-injury can predict AKI and MOF development in swine that underwent smoke inhalation and burn injury. For AKI prediction, the area under the ROC curve is 0.81 and, and the optimal cut-off value with a Youdex index of 36.41 ng/mL. The sensitivity and specificity using the optimal cut-off for predicting the outcome was 85.71%, and 75.00% respectively (**Figure 6A**). For MOF prediction, the area under the ROC curve is 0.89, and the optimal cut-off value is 36.41 ng/mL. The sensitivity and specificity for predicting MOF are 76.19% and 100%, respectively (**Figure 6B**). Traditional AKI parameters including creatinine and urine output did not correlate with AKI development at 12-hours post-injury (**Table 2**). The only risk factor identified that demonstrated association with AKI and MOF is MAP. However, both remained within their normal cut-off value of 100 mmHg physiological range and therefore are not clinically relevant.

Logistic regression analysis revealed that HMGB1 levels were significantly associated with AKI and MOF. At 12 hours post-injury, the animals with higher HMGB1 levels (≥ 36.41 ng/mL) compared to those with lower levels (<36.41 ng/mL) were associated with an 18-fold higher risk of AKI [OR:18.00 (2.33-387.0), *p*=0.02], and the animals with ≥ 36.41 ng/mL HMGB1 were associated with a 16-fold higher risk of MOF[OR:16.00(1.89-384.40), *p*=0.03] (**Table 3**).

**Table 3:** Factors associated with odds of AKI and MOF.

Univariate	AKI		MOF	
	OR (95% CI)	*p*-value	OR (95% CI)	*p*-value
HMGB1	1.04 (1.01-1.09)	0.02	1.05 (1.02-1.11)	<0.01
HMGB1 ≥36.41 VS. <36.41	18.00 (2.33-387.20)	0.02	16.00 (1.99-348.40)	0.03
SDC-1	1.26 (0.95-1.86)	0.17	1.34 (1.00-2.07)	0.08
C3a	1.00 (1.00-1.00)	0.77	1.00 (1.00-1.00)	0.58
Creatinine	5.72 (0.35-201.80)	0.35	3.95 (0.19-117.30)	0.78
Urine Output	1.01 (0.98-1.03)	0.73	1.00 (0.97-1.03)	0.51
MAP	0.96 (0.92-1.00)	0.02	0.95 (0.90-0.99)	0.03

AKI, acute kidney injury; MOF, multiple organ failure; HMGB1, high-mobility group box 1; SDC-1, syndecan-1; MAP, mean arterial pressure

## 4 Discussion

Early diagnosis of AKI and MOF allows for early and aggressive management of these complications, reducing the progression of deleterious diseases, and potentially improving mortality rates of trauma injuries (43). Few preclinical or clinical studies to-date have sought to develop prediction models for these complications in patients suffering from smoke inhalation and burn trauma. In our smoke inhalation and burn model, up to 20% of animals developed AKI at 24 hours as established by KIDGO criteria. This study also documented that 18% of the injured animals developed MOF at 24 hours after injury. Due to the nature of the injury, animals that developed MOF did so by combination of AKI and ARDS. Previous studies have shown that early diagnosis of acute kidney injury can be predicative of poor outcomes (44). Previous research revealed that early nephrology referral or diagnosis of AKI improves the clinical outcomes in patients with AKI as well (45, 46). We report in this study that HMGB1 levels correlate with high sensitivity to the onset of both AKI and MOF as early as 12 hours from the time of injury, whereas significant creatine rise cannot be determined until 24 hours or later.

Current guidelines define AKI as either a rise serum creatinine level or decrease in urine output (9). Although this is the current clinical standard it has several weaknesses: 1) recent research has indicated that serum creatinine is an insensitive biomarker that fails to reflect the acute change in renal function, instead correlating better with chronic kidney disease (47); 2) the level of serum creatinine varies with several factors not related to kidney function, including age, gender, muscle mass, diet, and medication use (48), creating the need to establish individual baselines for each patient; 3) serum creatinine is not a direct biomarker for tubular damage, but rather a marker reflecting glomerular filtration rate (GFR). It may be elevated in cases of renal hypo-perfusion even with structurally intact kidneys; and lastly 4) baseline values of serum creatinine are not known in many clinical situations, especially in a crisis situation, making objective assessments of the data difficult (49, 50). The use of urine output as a metric is non-specific (47) and can be affected by many variables, increasing complexity and decreasing the likelihood of successfully isolating the cause. Likewise, aggressive fluid resuscitation for critically ill causalities adds to variable complexity (47). Nonetheless, early recognition of AKI is vital to providing effecting damage control resuscitation to optimize care and prevent further injury by renal sensitive medications and interventions. Therefore, validating specific and sensitive biomarkers for AKI could close a critical gap in medicine, especially ones that present early enough to signal the need for interventions to optimize care (51). A 2020 consensus statement suggested that damage biomarkers should be integrated into the definition of AKI (18). Toward this end, many “novel” biomarkers have been proposed and continue to be investigated in the preclinical and clinical settings, including enzymes such as N-acetyl-β-glucosaminidase (NAG) (15), and α- and π-glutathione s-transferase [α-GST/π-GST] (52); proinflammatory mediators such as neutrophil gelatinase–associated lipocalin [NGAL] (13, 14), and IL-18 (16); proteins with other structures that might be elevated after tubular damage such as kidney injury molecule-1 [KIM-1] (53), liver-type fatty acid–binding protein [L-FABP] (54); and others such as retinol binding protein [RBP] (55), and angiotensinogen (56). Nevertheless, it is not clear if a single or combination of biomarkers are necessary to diagnose the complicated and multifactorial features of AKI (57–61). Additionally, research has attempted to characterize the sub-phenotype of patients with AKI, which may have different etiologies and outcomes, such as post-operative AKI (47) and/or sepsis-associated with AKI (62). However, there are few studies uncovering biomarkers associated with trauma-induced AKI. The detection of HMGB1 in the serum of injured animals in this study was performed by ELISA. In a laboratory setting, it typically takes 4-5 hours to finish the analysis and acquire results. However, we anticipate that many rapid tests (producing results on the order of 30 minutes or less) such as surface plasmon resonance (63), biosensors (64), and microfluidic devices (65) have been developed for measuring proteins/inflammatory mediators/cytokines in serum/blood samples that can be tailored or modified to detect HMB1 levels and/or activity in a rapid and reproducible manner to provide clinically relevant results informing AKI diagnoses.

The currently known primary mechanism that promotes trauma-induced AKI is ischemia from the reduction of blood flow. Ischemia results in cellular injury and organ dysfunction (66). As the kidney requires 20 to 25% blood flow to function properly, it is highly susceptible to ischemia induced injury, and the resulting vasoconstriction, endothelial damage, and inflammatory response activation (66, 67). However, additional factors beyond blood flow also likely contribute to AKI. Severe traumatic burn injury has two phases known as early and late burn-associated AKI (67). Early burn-related AKI results from acute tubular injury or tubular obstruction from the acute trauma. Late-related AKI is caused by hypovolemia, ischemia, and systemic inflammation (66). Reduced kidney perfusion can cause vessel damage (68), as reflected by a decrease in GFR and a multitude of related downstream physiological complications (69). Earlier studies of trauma AKI documented that inflammation recruited and induced the transmigration of neutrophils from vasculature into kidney interstitial tissue directly contributing to AKI (70). Ischemic injury also induces the release of oxygen-free radicals, which in turn cause oxidative stress. Oxidative stress causes the activation of many biochemical pathways, subsequently leading to inflammation and cell apoptosis, eventually causing tissue damage. Our study we documented many inflammatory cells infiltrated into the kidney tissue after smoke inhalation and burn injury. Moreover, we confirmed and extended previous studies documenting that increased cleaved caspase-3, which represents activation of apoptosis, was associated with aseptic trauma-induced AKI (71, 72), as well as in septic burns patients with AKI (73).

Trauma-induced ischemia activates the innate immune system through damage associated molecular patterns (DAMPS) as known to triggers multiple pro-inflammatory cascades, that increase renal dysfunction, and poor clinical outcomes (74). Recently, Mariano et al. reported the early dysregulated inflammatory responses in burn patients with AKI might be crucial that contributed to disease progress, whereas therapeutic approaches such as CytoSorb ^®^ aimed to control and/or reduce the early inflammatory responses will improve the clinical outcomes and survival (75). HMGB1, a DAMP molecule, plays a pivotal role in initiating and perpetuating the hyperinflammatory response after traumatic injury through activation of TLRs, RAGE, and other receptors (76, 77). Jiang et al. reported that in a rodent hemorrhage and septic AKI model, increased HMGB1 levels correlated with diagnosis of AKI (78). Huang et al. documented that the HMGB1 pathway is involved in LPS-induced AKI in a mouse model (79). Recently, Frelich et al. and colleagues evaluated 98 severely injured patients, and found that HMGB1 levels were significantly correlated with AKI (80). Even more, Zhao and colleagues by using knockout and inhibition strategies demonstrated intracellular HMGB1 is a potential target to enhance kidney regeneration and to improve long-term prognosis in AKI (81). Our results indicate that HMGB1 is associated with trauma-induced AKI from smoke-inhalation and burn. Furthermore, our data documents that HMGB1 can predict AKI development in a swine model of polytrauma. Logistic regression analysis determined that the cut off value for HMGB1 greater than 36.41 ng/mL increases the risk of AKI and MOF by 18-fold and 16-fold, respectively. Moreover, HMGB1 levels correlated strongly with standard AKI parameters BUN, creatinine, and myoglobin. Therefore, our data revealed that HMGB1 might serve as a promising prognostic biomarker for early prediction of trauma-induced AKI, garnering potential use in prehospital and prolonged field care settings.

Although AKI is a significant risk factor for mortality, further development of MOF significantly increases the risk (10). The Denver MOF score, for example, has a 48-hour window built in to incorporate the physiological changes under which organ failure is diagnosed as it is often delayed from onset of injury (66, 82), decreasing opportunities for life-saving treatment. Like AKI, the pathophysiology of MOF often involves a complex interplay of inflammatory mediators and cellular dysfunction across multiple organ systems. Recently, the potential for using DAMPs as physiological biomarkers of MOF to diagnose these conditions has been published (17, 19). Particularly, findings from our group and others identified HMGB1, as key DAMP that correlates well with organ failure at multiple layers, including 1) elevated serum HMGB1 levels are concurrent with ARDS in a swine polytrauma model (21); 2) HMGB1 contributes to systemic inflammation and MOF in patients with acute liver failure (ALF) (83); 3) HMGB1 levels highly correlate with systemic inflammatory response and injury severity scores (ISS) in combat casualties (84); and 4) HMGB1 inhibition significantly attenuated tissue damage in lung, brain and liver tissues as well as correlated with reduced mortality in a rat polytrauma model (84); and 5) HMGB1 plays a significant role in blast-induced MOF in a rat model. Overall, HMGB1 not only correlates to renal injury but also exerts systemic effects that can exacerbate dysfunction in other organs, especially in the lung, liver, and cardiovascular system. Further research is needed to determine if HMGB1 is the cause or the effect of organ damage following traumatic injury.

While promising, this study has potential limitations that include a small sample size (data from a population of 39 swine subjected to polytrauma), multiple treatment groups, and limited available tissue samples preventing measurement other markers of kidney injury such as NGAL. Although this study enrolled at least three different individual studies all reporting similar results, strengthening our observations, only a single mechanism of injury (polytrauma of smoke inhalation and burn injury) was used. Consequently, interventions did not correlate with the outcomes of AKI or MOF, based on the AKI and MOF distributions we observed in the three studies. Furthermore, earlier studies document that mesenchymal stem cells mitigate kidney injury following ischemic injury (85). This is consistent with the data that the saline group had increased amount of AKI compared to the MSC group. In addition, patients on ECMO support are known to have higher rates of AKI (86). However, several studies document that the increase in AKI is not associated with device support instead the lack of renal perfusion and inflammatory from the underlying condition (87). In addition, we noticed that the incidence of AKI, ARDS and MOF are different between the three protocols. We understand the differing protocols add variability that may limit the expounding of this study. Furthermore, in follow-up studies, it would be valuable to explore other traumatic injuries that have the propensity to cause AKI and MOF, such as hemorrhagic shock (88, 89) to test the correlation of HMGB1 to AKI and MOF in different mechanisms of injury. Lastly, we note that the incidence AKI and MOF are relatively low in this study by design at around 20%. The study’s primary design was to induce ARDS and correlates with clinical incidence in ARDS but nonetheless provides clinically relevant results for AKI as well.

## 5 Conclusion

We have identified a positive correlation between serum HMGB1 levels and the occurrence of AKI and MOF in a porcine model of smoke inhalation and burn injury with high sensitivity and specificity. Upon traumatic injury, subsequent biochemical and physiological processes recruit and activate/enroll HMGB1 activity to the injured tissue. This subsequent increase in HMGB1 levels strongly correlates and predicts the incidence of AKI and MOF in our model. This discovery has great prognostic potential for early prediction of AKI and MOF. Current diagnostic lead times have a basis of at least 24-hours by definition and kidney failure pathogenesis can take up to 7 days before current clinical definitions identify AKI, while our current model can predict the onset of AKI within 12 hours in a traumatic injury model.

The data presented here provides insight into the pathogenesis of renal injury and MOF, but also underscores its broader implications for early diagnostic and therapeutic application, particularly in trauma-induced organ failure and mortality. With timely intervention, patients with AKI significantly increase chance of survivability. Future research efforts should focus on further delineating the role of HMGB1 in AKI and MOF and translating these findings into clinical practice to optimize prehospital and combat casualty standards of care to improve patient outcomes and decrease mortality of these incidents.

## Data Availability

The original contributions presented in the study are included in the article/**Supplementary Material**. Further inquiries can be directed to the corresponding author.

## Author disclaimer

The views expressed in this article are those of the authors and do not reflect the official policy or position of the U.S. Army Medical Department, Department of the Army, DoD, or the U.S. Government. All research was conducted in compliance with the Animal Welfare Act, implementing Animal Welfare regulations, and the principles of the Guide for the Care and Use of Laboratory Animals. The Institutional Animal Care and Use Committee approved all research conducted in this study. The facility where this research was conducted is fully accredited by the AAALAC International.

## Supplementary material

Supplementary Figure 1 The dynamic changes of biomarkers in the blood circulation of animals in MOF and non-MOF groups. The dynamic changes of HMGB1 **(A)**, SDC-1 **(B)**, and C3a **(C)** in the serum in the groups of MOF (n=7) and Non-MOF (n=32) were analyzed by individual ELISA kits, and the data were presented as mean ± SEM. Statistical analyses were performed by linear mixed-effect model for repeated measures, and for the least square means of individual group comparisons. *, p<0.05, the value of individual time point of swine with MOF vs. Non-MOF; and †, p<0.05, for the least square means between the groups.

Supplementary Figure 2 The correlation of creatinine levels to myoglobin levels in injured swine after smoke inhalation and burn injury. At 24 hours post-injury, the correlation of the creatinine levels to the myoglobin levels were performed by Spearman’s rank correlation, and the data is presented with a coefficient(rs) and p-values. Significant correlations (p <0.05) are indicated by boldface type.
