# Supplementary figures and images for: An early HMGB1 rise 12 hours before creatinine predicts acute kidney injury and multiple organ failure in a smoke inhalation and burn swine model
Source: Front Immunol. 2024 Oct 29;15:1447597. doi: 10.3389/fimmu.2024.1447597 (PMC11554498; doi:10.3389/fimmu.2024.1447597)

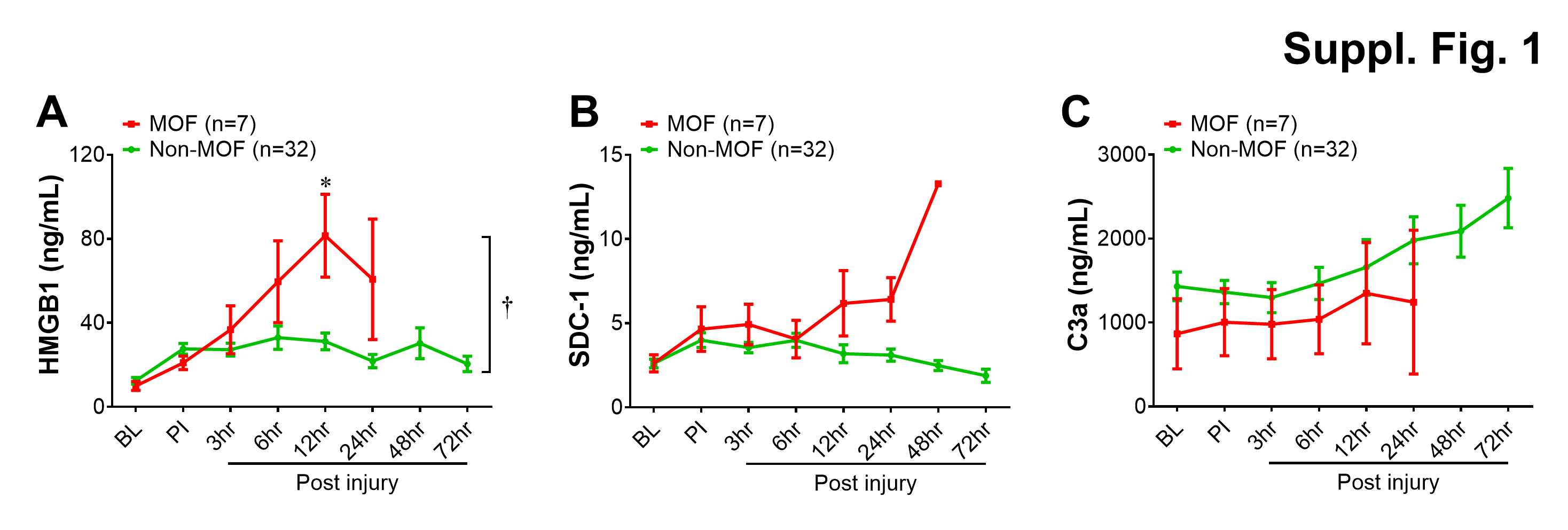

Supplement: Supplementary Figure 1 — The dynamic changes of biomarkers in the blood circulation of animals in MOF and non-MOF groups. The dynamic changes of HMGB1 (A), SDC-1 (B), and C3a (C) in the serum in the groups of MOF (n=7) and Non-MOF (n=32) were analyzed by individual ELISA kits, and the data were presented as mean ± SEM. Statistical analyses were performed by linear mixed-effect model for repeated measures, and for the least square means of individual group comparisons. *, p<0.05, the value of individual time point of swine with MOF vs. Non-MOF; and †, p<0.05, for the least square means between the groups. [file Image1.tif]

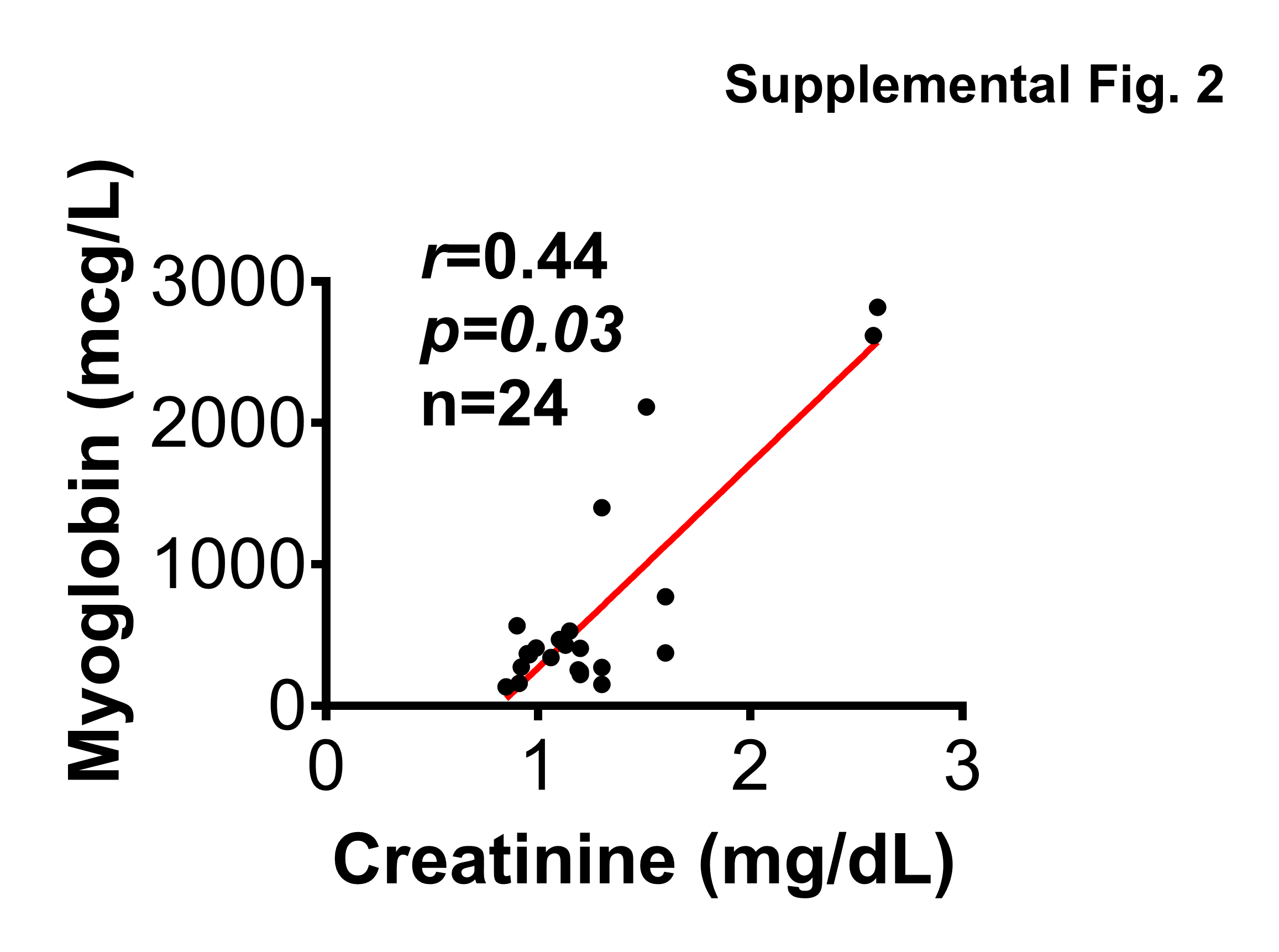

Supplement: Supplementary Figure 2 — The correlation of creatinine levels to myoglobin levels in injured swine after smoke inhalation and burn injury. At 24 hours post-injury, the correlation of the creatinine levels to the myoglobin levels were performed by Spearman’s rank correlation, and the data is presented with a coefficient(rs) and p-values. Significant correlations (p <0.05) are indicated by boldface type. [file Image2.tif]
